# Spatial phenotyping of nodular lymphocyte predominant Hodgkin lymphoma and T-cell/histiocyte-rich large B-cell lymphoma

**DOI:** 10.1038/s41408-024-01073-z

**Published:** 2024-05-31

**Authors:** Sheren Younes, Ajay Subramanian, Anum Khan, Shuchun Zhao, Michael Binkley, Yasodha Natkunam

**Affiliations:** 1 Department of Pathology, Stanford University School of Medicine, Stanford, CA USA; 2 Radiation Oncology, Stanford University School of Medicine, Stanford, CA USA; 3 Cell Sciences Imaging Facility, Stanford University School of Medicine, Stanford, CA 94305 USA

**Keywords:** Haematological cancer, Cancer microenvironment

## Abstract

Nodular lymphocyte-predominant Hodgkin lymphoma (NLPHL) is a rare lymphoma with sparse tumor B-cells and a favorable prognosis. Variant growth patterns of NLPHL, however, often show advanced stage, progression to T-cell/histiocyte-rich large B-cell lymphoma (THRLBCL) and a worse prognosis. We studied the tumor microenvironment (TME) of NLPHL and THRLBCL using highplex imaging and spatial profiling at the single cell level. Our findings show distinct differences in TME composition and spatial configuration that differ among typical and variant NLPHL and THRLBCL. Typical NLPHL show abundant helper T-cell subsets, while THRLBCL show abundant cytotoxic T-cells and macrophages. Tumor B-cell size and content is lowest in typical NLPHL, followed by variant NLPHL, and highest in THRLBCL, whereas an opposite trend characterized TME B-cells. CD4/CD8 double-positive T-cells are seen in all NLPHL but not in the majority of THRLBCL and are spatially distant from LP-cells and TFH-rosettes. The differences in macrophage/monocyte content in distinguishing NLPHL pattern E from THRLBCL is further corroborated in independent cohorts of cases. Our results validate the current approach to classification and in addition provide novel insights that could be leveraged to refine clinical management for patients with this spectrum of lymphomas.

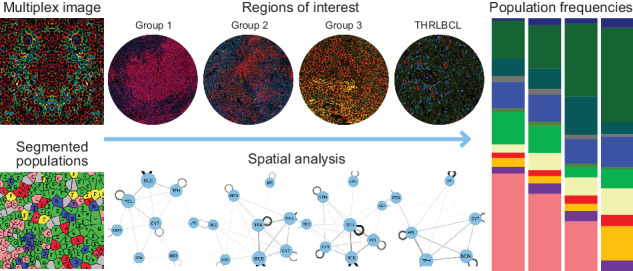

## Introduction

Nodular lymphocyte predominant Hodgkin lymphoma (NLPHL; also called nodular lymphocyte predominant B-cell lymphoma) is a rare neoplasm derived from germinal center (GC) B-cells [1–4]. Although the prognosis of NLPHL is favorable with a 10-year overall survival of >80%, up to 20–30% of patients experience recurrences and/or progression to large B-cell lymphoma, particularly, T-cell/histiocyte-rich large B-cell lymphoma (THRLBCL) [3–14]. The neoplastic lymphocyte-predominant (LP) cells of NLPHL form a small fraction of the tumor mass and are embedded in a rich tumor microenvironment (TME). We previously described six immunoarchitectural growth patterns of NLPHL which were found to correlate with disease recurrence and progression [15]. The prognostic relevance among the two “typical” and four “variant” growth patterns was subsequently confirmed and validated in independent adult and pediatric patient cohorts [6, 10, 16–18]. The recognition of variant growth patterns and their relationship to prognosis has prompted inquiries about the biologic basis that underlie these histologically-defined patterns, but has remained largely enigmatic.

De novo THRLBCL, unlike NLPHL, is associated with an aggressive clinical course, although an indolent course is seen in cases that arise from NLPHL [3, 6, 19, 20]. NLPHL and THRLBCL are increasingly recognized as a true biologic continuum due to striking similarities at the phenotypic and genetic levels. Despite extensive gene expression and mutational profiling studies, reproducible criteria to distinguish the variant patterns of NLPHL, particularly pattern E, from THRLBCL has yet to be established [6, 17, 21, 22]. Therefore, we studied the TME composition and spatial organization at the single cell level using CO-Detection by indEXing [CODEX® (rebranded as PhenoCycler™) [23–26] and a curated 21-antibody panel that was specifically designed to target tumor B-cells and immune cell subsets in the TME. By doing so, we gained insights into previously unexplored cellular interactions that could shed light on different growth characteristics associated with the biological behavior of NLPHL and THRLBCL.

## Methods

### Tissue samples

Formalin-fixed paraffin-embedded (FFPE) tissue from a total of 20 lymph node excision biopsies were retrieved from the Stanford Pathology archive. Ethics approval and consent to participate was obtained from the Stanford University Institutional Review Board in accordance with the Declaration of Helsinki and informed consent was obtained from all subjects (IRB Protocol IDs 11974 and 45081). All cases were reviewed by two pathologists (SY and YN). Sixteen NLPHL cases were selected based on immunoarchitectural patterns using slides stained for hematoxylin and eosin (H&E) and CD20 immunohistochemistry according to Fan et al. [15]. Of these, 8 cases contained typical patterns A and B, and 8 cases contained variant growth patterns, C, D and E; none had diffuse variant pattern F. In addition, four cases of THRLBCL were also selected which contained scattered tumor B-cells in a diffuse background. For analysis purposes, NLPHL cases were categorized into 3 groups: group 1, typical nodular growth patterns A and B; group 2, variant nodular growth patterns C and D; group 3, variant diffuse growth pattern E. THRLBCL cases comprised group 4. Details of the cases with the proportion of patterns are provided (Table 1).

**Table 1:** Clinicopathologic features of NLPHL and THRLBCL cases.

Codex ID	Analysis group	Diagnosis	Subtype (% variant patterns)	Age/Gender	Stage	Site of Biopsy (lymph node)	Management	Clinical course and outcome	Follow-up (years)
NC1	1	NLPHL	Typical	53/M	II	Inguinal	EBVP chemotherapy	Recurrence x1 24.5 years after diagnosis; currently ANED	26.5 years
NC2	1	NLPHL	Typical	38/F	I	Mesenteric	Complete excision alone	ANED	6.7 years
NC3	1	NLPHL	Typical	22/F	II	Cervical	R-CHOP chemotherapy	ANED	7.8 years
NC4	1	NLPHL	Typical	50/M	II	Cervical	Radiotherapy alone (30 Gy)	ANED	6.8 years
NC5	1	NLPHL	Typical	44/M	III	Mediastinal	R-CHOP	ANED	1.7 years
NC6	1	NLPHL	Typical	45/M	IV	Axillary	Prior chemo for THRLBCL (IV), NLPHL treated with radiotherapy alone (30 Gy)	ANED	6.1 years
NC7^a^	1	NLPHL	Typical	22/M	II	Cervical	Active surveillance after partial excision	A/recurrent disease	2.1 years
NC8	1	NLPHL	Typical	15/M	III	Cervical	Stanford V and radiotherapy	Recurrence x1 1 year after diagnosis; currently ANED	10 years
NV1	2	NLPHL	Variant (C, 70%)	7/M	II	Cervical	R-CHOP	ANED	3.1 years
NV3	2	NLPHL	Variant (C, 70%)	28/M	III	Submandibular	R-CHOP	ANED	3.8 years
NV5	2	NLPHL	Variant (C, 70%; D, 10%)	45/M	II	Mesenteric	R-CHOP	ANED	5.0 years
NV6	2	NLPHL	Variant (C, 30%)	69/F	II	Paratracheal	R-CHOP	Transformed to DLBCL; ANED	6.25 years
NV7	2	NLPHL	Variant (D, 20%)	67/M	IV	Cervical	R-CHOP	ANED	4.3 years
NV4	3	NLPHL	Variant (E, 80%)	67/M	IV	Cervical	R-CHOP	NA	NA
NV9	3	NLPHL	Variant (E, 60%; C, 10%; D, 10%)	15/M	II	Inguinal	R-CHOP	ANED	3 years
NV10	3	NLPHL	Variant (E, 60%)	M	II	Cervical	Stanford V	ANED	NA
TD1	4	THRLBCL	None	21/F	IV	Abdominal	R-EPOCH and radiotherapy (30 Gy)	ANED	5.5 years
TD5	4	THRLBCL	None	36/M	1	Submandibular	NA	NA	NA
TD6	4	THRLBCL	None	25/M	IV	Axillary	R-CHOP followed by autologous stem cell transplantation	DOD	2.1 years
TD7	4	THRLBCL	None	19/M	IV	Axillary	R-CHOP chemotherapy	NA	NA

^a^IGD + LP cells.

*EBVP* chemotherapy (epirubicin, bleomycin, vinblastine, prednisolone), *R-CHOP* chemotherapy (Rituximab, cyclophosphamide, doxorubicin, vincristine, prednisone), *Stanford V* (mechlorethamine, etoposide, vincristine, bleomycin, prednisone), *R-EPOCH* (Rituximab, etoposide, vincristine, doxorubicin, prednisone, cyclophosphamide), *ANED* alive with no evidence of disease, *DOD* died of disease, *NA* not available.

### Immunohistochemistry

Standardized automated immunohistochemistry (IHC) was performed on Ventana Benchmark Ultra (Roche Tissue Diagnostics, Tucson, AZ) or Leica BOND-III (Leica Biosystems, Buffalo Grove, IL). Commercially available antibodies were used (Supplementary Table S1).

### CODEX methodology

For CODEX antibody conjugations, 50 ug of carrier-free antibodies (Supplementary Table S1, Supplementary Fig. S1), were conjugated to DNA oligonucleotide barcodes using conjugation kits (Akoya Biosciences, CA). Antibodies were concentrated on a 50 kDa filter, equilibrated with filtration buffer, and sulfhydryl groups activated by incubating for 30 min at room temperature (RT) with reduction mix. Barcodes added to antibodies were incubated for 2 h at RT, eluted in 100 ul storage buffer, spun at 3000 × *g* for 4 min and stored at 4 °C until use. Conjugated antibodies were validated individually on positive control tissue and/or cell line pellets prior to the multiplex run (Supplementary Fig. S1). Fluorescent probes complementary to the DNA barcodes were added to the stained tissue for 5 min and imaged.

For CODEX antibody staining, 15 × 15 mm representative area selected from 4 µm FFPE sections were mounted on CODEX^R^ coverslips and stained with a 21-antibody cocktail. Coverslips were deparaffinized, rehydrated, and antigen retrieval performed using a pressure cooker for 20 min in 1X citrate buffer, pH 6.0 (NovusBio) followed by heating for 20 min at high pressure in 1X EDTA, pH 9.0 (Abcam). Autofluorescence was bleached as per protocol [27]. The antibody cocktail was added to coverslips and stained in a sealed humidity chamber at 4 °C overnight, washed, hydrated, and fixed according to manufacturer’s protocol (Supplementary Table S1).

For multicycle setup and imaging, coverslips mounted on Akoya’s custom stage was stained with Hoechst nuclear stain at a 1:2000 dilution in 1X CODEX buffer (Akoya). A 96-well plate for multicycle experiment was set up and different fluorescent oligonucleotides were added to a reporter stock solution (1:150 Hoechst stain and 1:12 dilution of assay reagent in 1X CODEX buffer) at a final concentration of 1:50 in a total of 250 ul per well. A blank cycle without fluorescent probes was performed at the start and end of the experiment to capture autofluorescence (Supplementary Table S2).

Automated image acquisition and fluidics was performed using Akoya’s software driver CODEX Instrument Manager (CIM, version 1.29) and the CODEX platform. Imaging was performed using a Keyence BZ-X810 microscope, fitted with a Nikon CFI Plan Apo 20X/0.75 objective. 11 z steps were acquired with the pitch set at 1.5 in the BZ-X software. Raw tiff files were processed using the CODEX Processor version 1.7.0.6 by Akoya to generate a large, stitched tiff file. Data processing included deconvolution, background subtraction and drift correction. Segmentation was performed using the nuclear signal by finding a local intensity maxima and then defining the radius for growth around this maxima. Threshold values for region growth are user-defined, and we used a radius of 5 and a size cut off factor of 0.1 to define the volume of a cell for accurate segmentation results. Segmentation accuracy was morphologically checked across ROIs by pathologists as shown (Supplementary Fig. S2). The segmentation algorithm accounts for spillover compensation of signal spillover from neighboring cells. Segmentation masks, spatial coordinates and marker intensities for all detected cells were then exported for further analysis (Supplementary Fig. S2).

For CODEX analysis, four representative regions of interest (ROIs) were digitally selected from each case by two pathologists to ensure NLPHL patterns were adequately represented (Supplementary Fig. S3). The selected ROIs were comparable across all 20 NLPHL and THRLBCL cases and the number of cells per ROI ranged between 10,431–65,884, mean +/− SD of 33965.7+/− 14331.6. Multiplex Analysis Viewer (MAV, Akoya), an ImageJ plugin, was used to visualize and validate marker expression, determine segmentation accuracy, extract ROI’s and gate data for further analysis.

Clustering was performed in R using the Seurat package for unsupervised clustering [28–33]. Populations were defined based on coexpression of defining markers and confirmed by absence of other non-specific markers to diminish the effect of overlapping signals. Post-clustering annotation of cellular phenotypes was performed by the two pathologists.

Spatial analysis was performed using MAV, which defines interactions based on spatial proximity. A distance of 3–30 μm was used to calculate the number of interacting cells, which is a ratio that defines the likelihood that cells within a 3–30 μm radius from a particular cell are interacting with each other meaningfully compared to doing so by chance. An interacting cell as defined by MAV is any cell that falls within the predefined spatial distance range. To better define TME cells for study, we broadly grouped cell populations by phenotype and by their activation status as defined in Table 2.

**Table 2:** Cell populations analyzed in NLPHL and THRLBCL.

Cell types (Z-scores)	Phenotype	NLPHL			THRLBCL
		Group 1	Group 2	Group 3	
B-cell subsets					
Tumor B^a^	CD20/BCL6	−0.47	−0.35	0.36	1.10
TME B	CD20/IgD	0.77	0.28	−0.38	−1.61
T-cell subsets					
Helper T	CD3/CD4	0.50	0.15	−1.06	−0.40
Activated Helper T	CD3/CD4/LAG3/CD69	−0.14	−0.00	1.01	−0.48
Follicular Helper T (TFH)	CD3/CD4/PD1/BCL6	0.16	0.32	−0.05	−0.68
Activated TFH (aTFH)	CD3/CD4/PD1/BCL6/LAG3/CD69	−0.55	−0.37	0.52	1.18
Cytotoxic T-cells	CD3/CD8	−0.05	−0.04	−0.21	0.29
Activated cytotoxic T	CD3/CD8/LAG3/CD69	−0.39	0.19	0.03	0.50
Regulatory T-cells (T-reg)	CD3/CD4/FOXP3	−0.31	0.14	0.12	0.34
CD4/CD8 double-positive T (DPT)	CD3/CD4/CD8	0.17	0.24	−0.09	−0.59
Macrophage/Monocyte subsets					
Macrophages	CD68	−0.21	−0.29	−0.03	1.02
Monocytes	CD68/MPO/CD14	−0.54	0.12	0.18	0.79

^a^Lymphocyte Predominant (LP)-cells in NLPHL, and large atypical B-cells in THRLBCL

### QuPath analysis for tumor cell size and macrophage/monocyte counts

To assess tumor cell size, MEF2B immunohistochemistry (polyclonal, 1:200 dilution; Sigma-Aldrich, St Louis, MO), which targets tumor cell nuclei only, was employed. We utilized QuPath (V0.4.4; https://qupath.github.io/) to quantify nuclear area and perimeter of MEF2B positive tumor cells. Two cores from each case were employed in constructing a TMA, which is utilized for measuring nuclear diameters. QuPath was employed to semiquantitatively evaluate the proportion of positive macrophage/monocyte markers including PU.1, CD163, and CD14. Independent cohorts of 15 NLPHL pattern E cases and 14 THRLBCL cases were utilized. Two representative cores were selected from each case and the median positive percentages were calculated.

### Statistical analysis

R version 4.1.2 in R server and R console environments were used to quantify cell population abundance per region/case. To consolidate values across regions within each case, means were computed. For patterns, values were averaged across regions that matched the pattern. Significance testing at the region level was performed using rank-sum Wilcoxon *t* tests. The abundances were normalized to mean zero and unit variance. For visualization, R packages ‘ggplot2’ for bar and box plots and ‘ComplexHeatmap’ for heatmaps were used. To explore interactions among cell populations, cell counts and interaction matrices describing each population within each region from MAV were extracted. To account for differences in the cell population sizes, we corrected each interaction for the total number of cells involved in the interaction. We conducted rank-sum Wilcoxon *t* tests for testing the significance of these interactions. For visualizing interactions, we used the ‘chordDiagram’ R package and the ‘corrplot’ R package. All *P*-values were two-tailed and considered significant at *P* < 0.05. IBM SPSS Statistics (version 29) was utilized to compute the median expression levels of macrophage markers and nuclear perimeter. Statistical analysis was performed using the Mann–Whitney U test to compare medians and determine statistical significance.

## Results

### Composition and abundance of tumor B-cells, and TME B-cells and T-cells, vary in NLPHL and THRLBCL

**Fig. 1:**
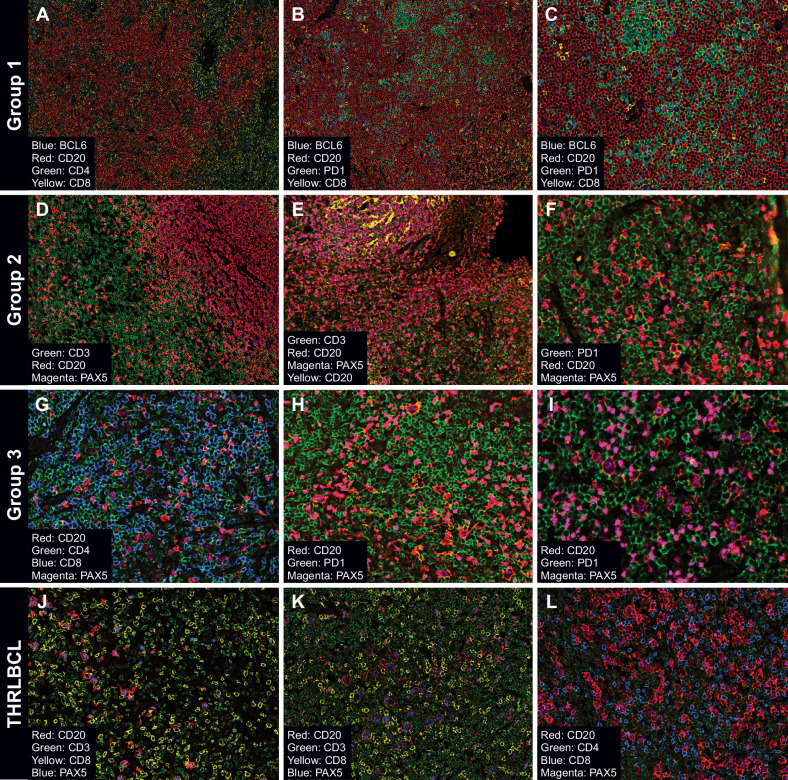
Typical examples of multiplex images of NLPHL and THRLBCL generated by CODEX. Multiplex images of LP (NLPHL groups) and tumor cells (THRLBCL) and the surrounding tumor microenvironment cell subsets are shown as follows: Group 1, nodular patterns A and B (**A**–**C**); Group 2, nodular patterns C and D (**D**–**F**); Group 3, diffuse pattern E (**G**–**I**); and THRLBCL (**J**–**L**). Selected combinations of markers are shown in each nodular or diffuse immunoarchitectural configuration to highlight the range of patterns seen in the different study groups.

**Fig. 2:**
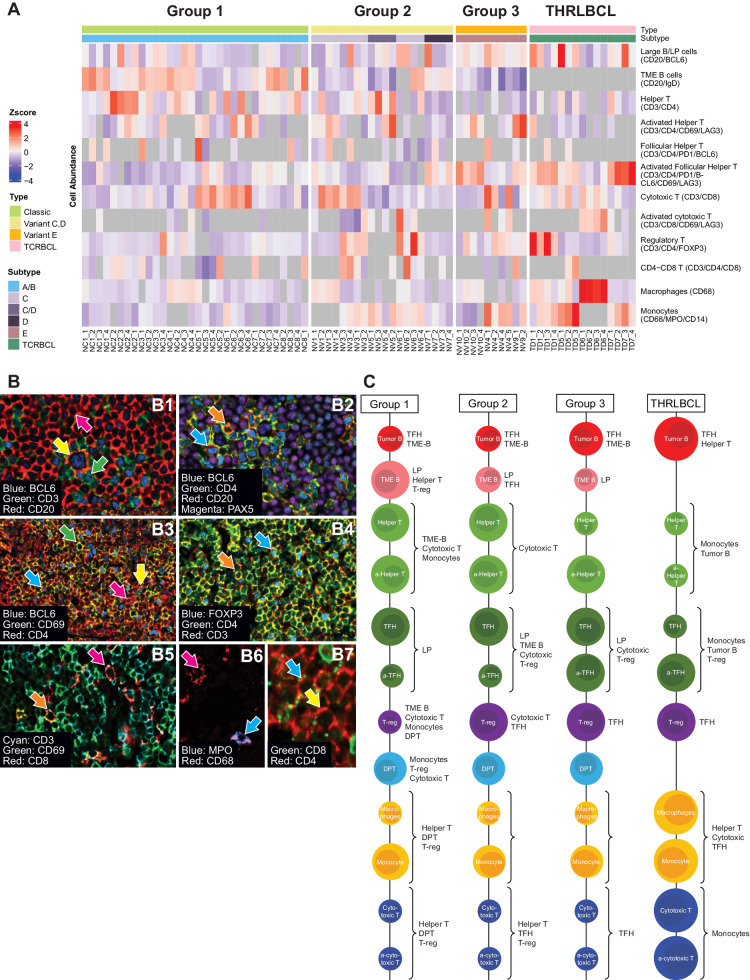
Cellular populations and their relative abundance as defined by co-expression of markers. Heatmap of population frequencies in ROIs clustered by NLPHL groups and THRLBCL (**A**). Multiplex images show cellular populations stained with identifying markers in NLPHL and THRLBCL (**B**); arrows indicate the following cell types: panel B1 shows LP cells (CD20/BCL6 in yellow), TFH cells (CD3/CD4/PD1/BCL6 in green), and TME B cells (CD20/IGD in red); panel B2 shows TH cells (CD3/CD4 in orange) and TFH cells (green); panel B3 shows TH cells (orange), activated TH (CD3/CD4/CD69/LAG3 in green), TFH (blue), and activated TFH (CD3/CD4/PD1/BCL6/CD69/LAG3 in yellow); panel B4 TH cells (red), activated TH cells (green), TFH (yellow), activated TFH (blue); panel B4 shows TH cells (orange) and T-regs (CD3/CD4/FOXP3 in blue); panel B5 shows cytotoxic T-cells (CD3/CD8 in red), activated cytotoxic T-cells (CD3/CD4/CD69/LAG3 in yellow); panel B6 shows macrophages (CD68 in red) and monocytes (CD68/MPO/CD14 in blue); and panel B7 shows CD4/CD8 DPT-cells (blue). Diagram displays the relative abundance of cellular populations and the most significant cell-cell interactions in groups 1, 2, and 3 NLPHL, and THRLBCL (**C**).

The four analysis groups were evaluated by the 21-marker CODEX panel (Table 1, Fig. 1A–L). Cell types and their functional status were defined and visualized in each case and each ROI per case (Table 2, Fig. 2 and Supplementary Fig. S4). Z-scores allowed comparison of the proportion of each cellular phenotype in relation to the overall number of cells within each ROI (Table 2 and Supplementary Table S3; Fig. 2 and Supplementary Fig. S4).

Tumor B-cell numbers were highest in THRLBCL followed by groups 3, 2 and 1 NLPHL (groups 3 vs 1, *p* = 0.009; and groups 3 vs 2, *p* = 0.05). In contrast, NLPHL showed significantly higher TME B-cells in groups 1 vs 2 and 3 (*p* = 0.003 and <0.0001, respectively). The sparse TME B-cells in THRLBCL could not be detected by the clustering algorithm (Fig. 2A–C).

T-cell subsets showed the following distributions: T-helper (TH) cells (CD3/CD4) and activated TH cells (CD3/CD4/LAG3/CD69) were more abundant in the NLPHL TME compared with the THRLBCL TME (for TH, groups 1 vs 3 and THRLBCL, *p* = 0.04 and *p* = 0.025, respectively; for activated TH, group 3 vs THRLBCL, *p* = 0.01). In contrast, regulatory T-cells (T-regs, CD3/CD4/FOXP3) were low in group 1 and showed a gradual increase in the TME of groups 2, 3 and THRLBCL. TFH cells (CD3/CD4/PD1/BCL6) were more abundant in all groups of NLPHL relative to THRLBCL. Interestingly, activated TFH cells (CD3/CD4/PD1/BCL6/LAG3/CD69) showed a reverse relationship with significantly lower abundance in groups 1 vs 3 and THRLBCL (*p* = 0.001 and <0.0001, respectively), and groups 2 vs 3 and THRLBCL (*p* = 0.03 and 0.002, respectively). In contrast, cytotoxic (CD3/CD8) and activated cytotoxic (CD3/CD8/LAG3/CD69) T-cells were abundant in THRLBCL relative to all groups of NLPHL (Table 2, Fig. 2A–C and Supplementary Fig. S4).

Additionally, CD4/CD8 double-positive T (DPT)-cells were detected in all NLPHL cases (group 1, 7 of 8 cases, 87.5%; group 2, 4 of 5 cases, 80%; and group 3, 2 of 3 cases, 66.7%). Among THRLBCL, only 1 of 4 (25%) cases showed CD4/CD8 DPT-cells (Table 2, Fig. 2A–C and Supplementary Fig. S4).

### Macrophage/monocyte subsets are increased in THRLBCL compared to NLPHL

**Fig. 3:**
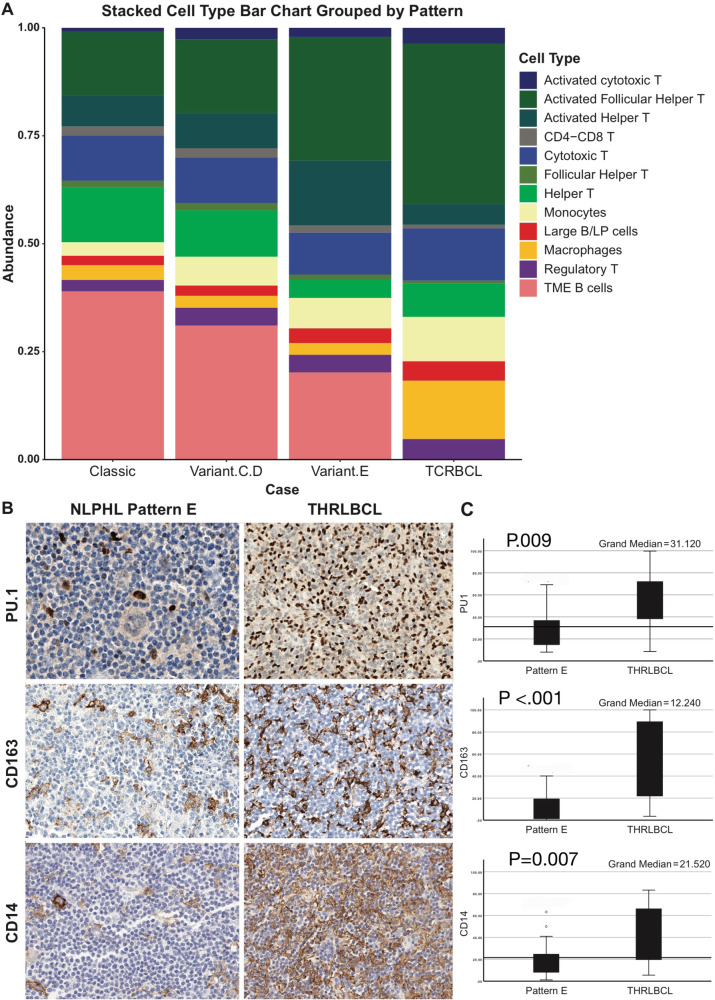
Relative abundance of macrophages and monocytes as assessed by CODEX and Immunohistochemistry. Stacked bar plot with frequencies of cellular populations grouped by disease categories show the most striking difference in macrophages and monocytes (**A**). Immunohistochemistry using individual macrophage/monocyte markers in independent cohorts of NLPHL pattern E and THRLBCL show increased staining for PU.1 (**B**, top row), CD163 (**B**, middle row), and CD14 (**B**, bottom row). Bar plots show summary of QuPath derived quantification of NLPHL pattern E and THRLBCL (**C**).

Both macrophages (CD68) and monocytes (CD68/MPO/CD14) were more abundant in THRLBCL compared to all NLPHL groups (Table 2, Figs. 2A–C and 3A). There were some comparisons where THRLBCL showed a statistically significant abundance in macrophages (THRLBCL vs group 2, *p* = 0.036), and monocytes (THRLBCL vs. groups 1 and 2, *p* ≤ 0.001 and 0.017, respectively) (Supplementary Table S4; Fig. 3B, C).

Overall, our analysis showed that macrophage/monocyte content had the highest differences among all cell types analyzed in NLPHL groups and THRLBCL (Fig. 3A–C). We then investigated whether macrophage/monocyte content could aid in the separation of NLPHL pattern E from THRLBCL, which is well known to be a difficult diagnostic boundary. Using individual immunohistochemical staining for PU.1, CD163 and CD14, and QuPath image analysis, we investigated independent cohorts of NLPHL pattern E (15 cases) and THRLBCL (14 cases). Our results show that all three markers are significantly increased in THRLBCL compared to NLPHL pattern E, which substantiates the CODEX data (Supplementary Table S4; Fig. 3B, C).

### NLPHL and THRLBCL show distinct spatial organization

**Fig. 4:**
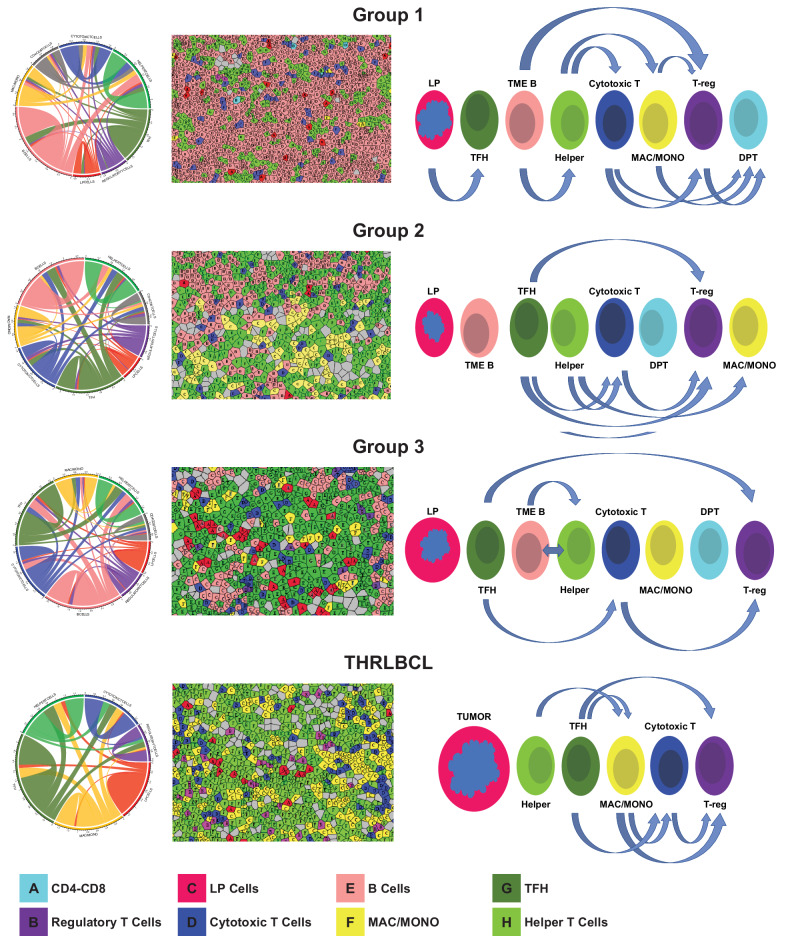
Spatial interactions and cellular phenotypes in NLPHL and THRLBCL. Spatial interactions by NLPHL groups 1, 2 and 3 as well as THRLBCL are depicted by Circos plots, which represent the sum of all ROIs in each of the groups 1, 2 and 3 NLPHL, and THRLBCL (**A**). The corresponding color matched Voronoi diagrams of clustered cell types represent examples of single ROIs in each group (**B**). Diagram of cellular interactions demonstrate the spatial configuration of populations and specific cell-cell interactions between LP/tumor cells and the tumor microenvironment in groups 1, 2 and 3 NLPHL, and THRLBCL (**C**).

All spatial interactions among the cell types that were defined by the 21-marker CODEX panel in NLPHL groups and THRLBCL were calculated and illustrated (Table 3 and Supplementary Tables S5–9; Fig. 4 and Supplementary Figs. S5, 6). Specific spatial interactions of the cellular populations analyzed are described in detail below.

**Table 3:** Interaction counts between LP cells and TME components.

Cell type	NLPHL			THRLBCL
	Group 1	Group 2	Group 3	
Tumor B-cells	0.26	0.24	0.29	0.51
TME B-cells	0.065	0.062	0.047	NA
Helper T-cells	0.056	0.045	0.047	0.095
Follicular helper T-cells (TFH)	0.094	0.058	0.079	0.075
Cytotoxic T-cells	0.046	0.034	0.044	0.04
Regulatory T-cells (T-reg)	0.029	0.029	0.029	0.027
CD4/CD8 T-cells (DPT)	0.027	0.034	0.034	NA
Macrophages/monocytes (mac/mono)	0.039	0.023	0.039	0.05

Tumor B-cells showed differences in spatial localization among the NLPHL groups. In group 1, LP cells had the strongest interaction/spatial proximity to TFH cells followed by TME B-cells but did not interact strongly with cytotoxic or TH cells. In group 2, TME B-cells were more proximal to LP cells than TFH cells. Furthermore, there was a statistically significant stronger interaction between LP cells and TFH cells in group 1 compared to group 2 NLPHL (*p* = 0.04). In group 3, TFH cells showed the most spatial proximity to LP cells, followed by TH cells and TME B-cells. Macrophages and cytotoxic T-cells were spatially distant from LP cells. Finally, in THRLBCL, TH cells were found to show the highest proximity to tumor B-cells, followed by TFH cells, and then macrophages and cytotoxic T-cells (Table 3 and Supplementary Tables S5–9; Fig. 4 and Supplementary Figs. S5, 6).

TME B-cells were spatially proximal to TH and T-reg cells in typical NLPHL with the highest level of TH-TME B-cell proximity in group 1 compared to groups 2 and 3 (*p* = 0.002 and 0.035, respectively). Similarly, T-regs interacted strongly with TME B-cells in group 1 compared to groups 2 and 3 (*p* = 0.002 and 0.013, respectively).

TH and cytotoxic T-cell interactions showed significant differences in nodular versus diffuse configurations across NLPHL and THRLBCL. There was a notable, significantly stronger interaction between TH and cytotoxic T-cells in group 1 compared to group 3 and THRLBCL (*p* = 0.002 and 0.017, respectively). Similarly, TH-cytotoxic T-cell interactions were more pronounced in group 2 compared to group 3 and THRLBCL (*p* = 0.012 and 0.03, respectively). In addition, TH-macrophage interactions were significantly higher in group 1 compared to groups 2 and 3 (*p* = 0.06 and <0.0001, respectively), and between group 2 and group 3 (*p* = 0.012).

T-regs showed strong interactions with TME B-cells, macrophages, cytotoxic T-cells, and CD4/CD8 DPT-cells in group 1. In contrast, T-regs and TFH populations showed stronger interactions that were statistically significant in groups 2 and 3, as well as THRLBCL in comparison to group 1 (*p* = 0.004, 0.002, and 0.02, respectively). The overall abundance of T-regs was, however, the lowest in group 1 among all cases evaluated. Our spatial analysis data takes into consideration the number of interactions between populations in relation to the number of cells in each of the studied groups (Table 3 and Supplementary Tables S5–9; Fig. 4 and Supplementary Figs. S5, 6).

Cytotoxic T-cells exhibited closer proximity to macrophages in THRLBCL in comparison to groups 1, 2, and 3 (*p* > 0.05, 0.019, and 0.002, respectively). Furthermore, cytotoxic T-cells and TFH cells were found to have the strongest spatial relationship in groups 2 and 3 as well as THRLBCL compared to group 1 (*p* = 0.013, >0.05, and 0.019, respectively) and was least consequential in THRLBCL. Moreover, cytotoxic T-cells and T-regs showed a significant spatial proximity in groups 1 and 2 compared to THRLBCL (*p* = 0.0006 and 0.006, respectively).

CD4/CD8 DPT-cells were spatially distant from the vicinity of LP cells and TFH-rosettes where most TME B-cell interactions with LP cells were seen. The DPT-cells showed stronger interaction with cytotoxic T-cells in groups 1 vs 2 (*p* = 0.047). These DPT-cells also interacted with macrophages and T-regs in group 1 compared to group 3 (*p* = 0.02 and 0.03, respectively). Overall, cytotoxic T-cells, T-regs, CD4/CD8 DPT-cells and macrophages were spatially distant from LP cells when compared with TME B-cells and TFH cells in all groups of NLPHL (Tables 2, 3 and Supplementary Tables S3, S5–9; Figs. 2, 4 and Supplementary Figs. S5, 6).

Spatial proximity between macrophages and T-regs were prominent in group 1 compared to group 3 and THRLBCL (*p* = 0.003 and 0.048, respectively). In contrast, the proximity between macrophages and TFH cells was the highest in THRLBCL compared to groups 1, 2 and 3 NLPHL (*p* ≤ 0.0001, 0.0001, and 0.001, respectively).

### Tumor cell size differs in NLPHL and THRLBCL

Tumor cell nuclear area and perimeter showed a gradual increase from groups 1 to 2 to 3 NLPHL (*p* < 0.001 for groups 3 vs 2, groups 3 vs 1 and groups 2 vs 1, *p* < 0.001 for nuclear area and *p* < 0.001 for groups 3 vs 1 and groups 3 vs 1; and *p* = 0.018 for groups 3 vs 2 for nuclear perimeter). Both nuclear measurements were smaller in THRLBCL compared to group 3 NLPHL (*p* < 0.001); however, nuclear size of THRLBCL remained higher than both groups 1 and 2 NLPHL (*p* < 0.001). (Supplementary Table S10; Supplementary Fig. S7).

## Discussion

NLPHL and THRLBCL are considered to represent different endpoints of the same disease spectrum [6]. Gene expression profiling, copy number alterations, and mutational profiling studies have further underscored the extensive overlap between these entities [6, 27, 34–40]. Although the drivers of transformation from NLPHL to THRLBCL remain largely unknown, the differences in prognosis highlight the critical need for more robust criteria to distinguish NLPHL, especially pattern E, from THRLBCL. Our results, in a small albeit carefully selected cohort of NLPHL and THRLBCL, provide valuable insights to understand tumor-TME dynamics and a baseline for further work to explore their clinical implications.

Both NLPHL and THRLBCL have sparse tumor B-cells; however, our data clearly show an ordered abundance of tumor B-cells from least abundant in typical patterns, followed by variant patterns of NLPHL, and most abundant in THRLBCL. Our spatial data further captured substantial differences in the TME, both proximal to and distant from tumor B-cells.

Nodular NLPHL patterns had increased TME B-cells and TH cells but decreased activated TH cells compared to NLPHL pattern E and THRLBCL. A strong TH and cytotoxic T-cell interaction was seen in nodular groups, whereas interaction between TH and tumor cells was more pronounced in diffuse pattern cases. Since CD4 + T cells produce pro-inflammatory cytokines that lead to recruitment and activation of innate effector cells [4], the observed strong interactions between TH cells with cytotoxic T-cells and macrophages may contribute to the superior prognosis of typical NLPHL. The distinct cellular interactions noted exclusively in group 1 NLPHL indicate that it is not the abundance of TME components but rather the interactions among cell populations that is likely to underlie the histologic and associated prognostic differences.

Notably, TFH abundance and activation status was higher in all NLPHL groups compared to THRLBCL; activated TFH cells were more abundant in diffuse compared to nodular patterns of NLPHL. Spatial analysis showed that TFH cells were the closest to LP cells in typical NLPHL and confirm prior observations that TFH-rosettes, which form an immunologic synapses, were most abundant in typical NLPHL [40–42], less frequent in variant patterns, and rare in THRLBCL [6, 17, 43, 44].

The alignment of TH and TFH cell activation as revealed by increased PD1 and CD69 expression in diffuse pattern E and THRLBCL in our cohort is of interest. CD69, a marker of T-cell activation, has also been detected by flow cytometry in NLPHL [45]. Upon activation, PD1 and CD69 are simultaneously expressed in activated TFH cells and induce an immunosuppressive microenvironment by induction of T-cell exhaustion [46]. In contrast to TH cells, the interaction between T-regs and TFH cells was the least in group 1 NLPHL compared to the other groups.

The separation of variant NLPHL patterns, particularly, pattern E from THRLBCL is difficult and sometimes impossible in routine clinical practice using currently available diagnostic tools [47]. Our results revealed insights that could potentially offer guidance for clinical management and explain clinical behavior. Specifically, our data yielded three unique TME features that distinguish NLPHL from THRLBCL: (1) at least a two-fold increase in activated TH and TFH cells in all NLPHL groups compared to THRLBCL; (2) a remarkable abundance of macrophage/monocyte subsets and cytotoxic T-cells in THRLBCL relative to NLPHL; and (3) detection of CD4/CD8 DPT-cells in NLPHL but not in the majority of THRLBCL.

The differences in macrophage/monocyte content among NLPHL and THRLBCL detected by CODEX, led us to use single immunohistochemical stains with image analysis to confirm that NLPHL pattern E has distinctly fewer macrophages and monocytes compared to THRLBCL. We also found that macrophages form the bulk of this difference. These findings have clinical implications and offer a potential tool for discriminating NLPHL pattern E from THRLBCL, especially when image analysis is used. Given the lack of specificity of CD68 when used as a single immunohistochemical marker in tissue sections, we employed PU.1, CD163 and CD14 for our analysis. These markers are widely used in clinical practice and could be combined with automated immunohistochemistry platforms that have begun to incorporate image analysis for quantitative read-outs. Once validated in larger cohorts of cases, macrophage content assessment using image analysis could prove to be of significant clinical benefit in the diagnosis of NLPHL and THRLBCL. Prior work in the field has shown that high macrophage content in NLPHL and THRLBCL correlates with the acquisition of a tolerogenic TME and poor prognostic factors including a lower complete remission rate, bulky disease, and variant growth patterns [17, 48–51]. Therefore, measuring macrophage content has added value in diagnosis, although given the substantial overlap in cellular composition in NLPHL and THRLBCL, careful correlation with additional clinicopathologic features is imperative for definitive diagnosis. In addition to macrophages, difference in cytotoxic T-cell content and interactions with LP cells, which were stronger in NLPHL pattern E compared to other NLPHL groups, may influence responses to immune therapy and prognosis as shown in other cancer types [52].

Another difference between NLPHL and THRLBCL comes from CD4/CD8 DPT-cells detected by unsupervised clustering in 67–77% of NLPHL, with no significant difference among NLPHL groups. These DPT-cells were previously detected in NLPHL by flow cytometry [53]. The DPT-cells showed no discernible interaction with LP cells and were spatially distant from LP cells and TFH rosettes. In group 1 NLPHL only, DPT-cells interacted with cytotoxic T-cells, macrophages, and T-regs. Given that only a single case of THRLBCL had DPT-cells, further investigation of a larger cohort of THRLBCL is warranted to confirm these observations.

To the best of our knowledge, our study is the first description of the composition and spatial configuration of TH, TFH, Tregs, and their activated counterparts as well as macrophage/monocyte subsets in NLPHL and THRLBCL. This annotation was made possible by the simultaneous use of LAG3 and CD69 to define activation status among T-cell subsets along with other subset-specific markers for other lineages. Their individual functional contributions to the pathogenesis and progression of NLPHL and THRLBCL, however, requires further study.

Our data also demonstrated a gradual increase in tumor cell size from the smallest tumor cells in typical NLPHL and largest in variant NLPHL. In previous studies using 3-dimentional confocal microscopy, nuclear volume and cell volume of THRLBCL tumor cells was found to be increased in comparison to LP cells of NLPHL [53]. In addition, in a cohort of 152 NLPHL cases, larger mean nuclear size as measured by image analysis using QuPath software correlated with variant growth patterns [54]. Although the exact measurements are slightly different from our study, the overall findings show variation in LP cell size which is increased in variant pattern NLPHL compared with typical NLPHL. In addition, the graded increase in tumor B-cell size in our cohort aligns with the presence or absence of nodularity in the overall immunoarchitecture of NLPHL and THRLBCL. Although nodularity and FDC meshworks were not formally evaluated in this study, their influence on tumor cell size may have implications for lymphoma progression and capacity for dissemination in the less confined architectural configurations that characterize variant NLPHL and THRLBCL in contrast to typical NLPHL.

In conclusion, by deeply profiling the cellular composition and spatial organization of NLPHL and THRLBCL, we corroborate known associations and discover unrecognized novel cellular interactions. These data offer insights regarding abundance, functional status, and spatial distribution of specific cell populations at the single-cell level in whole tissue sections that were carefully annotated by pathologists. Overall, this study provides a validation of the current approach to classification of NLPHL and its variant growth patterns, and THRLBCL. In addition, cell type abundance measures were leveraged to validate distinct differences in macrophage/monocyte subsets between NLPHL pattern E and THRLBCL, which is a difficult diagnostic boundary in clinical practice. Furthermore, these data provide the groundwork for further study of larger retrospective and prospective cohorts of clinically well-annotated NLPHL and THRLBCL, to confirm and validate the diagnostic utility and clinical implications of these findings. Additionally, more detailed functional subset marker panels could be utilized to differentiate activated TH cells including TH1, TH2 and TH17, as well as follicular dendritic cells in the transition from NLPHL to THRLBCL. Our approach also provides a framework to address the broader paradigm of architectural dismantling of the lymph node niche and its substructures including changes in the secondary follicle in neoplasia such as follicular lymphoma, and in non-neoplastic conditions such as progressive transformation of germinal centers and Castleman disease, among others.

### Supplementary information

Supplemental figures

Supplemental Tables

## Data Availability

The datasets generated and analyzed during the current study are available from the corresponding author on reasonable request.

## References

[1] Morton LM, Wang SS, Devesa SS, Hartge P, Weisenburger DD, Linet MS (2006). Lymphoma incidence patterns by WHO subtype in the United States, 1992–2001. Blood.

[2] Teras LR, DeSantis CE, Cerhan JR, Morton LM, Jemal A, Flowers CR (2016). US lymphoid malignancy statistics by World Health Organization subtypes. CA Cancer J Clin.

[3] Alaggio R, Amador C, Anagnostopoulos I, Attygalle AD, Araujo IBO, Berti E (2022). The 5th edition of the World Health Organization Classification of Haematolymphoid Tumours: Lymphoid Neoplasms. Leukemia.

[4] Al-Mansour M, Connors JM, Gascoyne RD, Skinnider B, Savage KJ (2010). Transformation to aggressive lymphoma in nodular lymphocyte-predominant Hodgkin’s lymphoma. J Clin Oncol.

[5] Biasoli I, Stamatoullas A, Meignin V, Delmer A, Reman O, Morschhauser F (2010). Nodular, lymphocyte-predominant Hodgkin lymphoma: a long-term study and analysis of transformation to diffuse large B-cell lymphoma in a cohort of 164 patients from the Adult Lymphoma Study Group. Cancer.

[6] Hartmann S, Döring C, Jakobus C, Rengstl B, Newrzela S, Tousseyn T (2013). Nodular Lymphocyte Predominant Hodgkin Lymphoma and T Cell/Histiocyte Rich Large B Cell Lymphoma—Endpoints of a Spectrum of One Disease?. PLoS One.

[7] Eyre TA, Gatter K, Collins GP, Hall GW, Watson C, Hatton CS (2015). Incidence, management, and outcome of high-grade transformation of nodular lymphocyte predominant Hodgkin lymphoma: Long-term outcomes from a 30-year experience. Am J Hematol..

[8] Eichenauer DA, Plütschow A, Fuchs M, von Tresckow B, Böll B, Behringer K (2015). Long-Term Course of Patients With Stage IA Nodular Lymphocyte-Predominant Hodgkin Lymphoma: A Report From the German Hodgkin Study Group. J Clin Oncol.

[9] Kenderian SS, Habermann TM, Macon WR, Ristow KM, Ansell SM, Colgan JP (2016). Large B-cell transformation in nodular lymphocyte-predominant Hodgkin lymphoma: 40-year experience from a single institution. Blood.

[10] Shankar AG, Roques G, Kirkwood AA, Lambilliotte A, Freund K, Leblanc T (2017). Advanced stage nodular lymphocyte predominant Hodgkin lymphoma in children and adolescents: Clinical characteristics and treatment outcome—A report from the SFCE & CCLG groups. Br J Haematol..

[11] Marks LJ, Pei Q, Bush R, Buxton A, Appel B, Kelly KM (2018). Outcomes in intermediate-risk pediatric lymphocyte-predominant Hodgkin lymphoma: A report from the Children’s Oncology Group. Pediatr Blood Cancer..

[12] Binkley MS, Rauf MS, Milgrom SA, Pinnix CC, Tsang R, Dickinson M (2020). Stage I-II nodular lymphocyte-predominant Hodgkin lymphoma: A multi-institutional study of adult patients by ILROG. Blood.

[13] Eichenauer DA, Plütschow A, Fuchs M, Sasse S, Baues C, Böll B (2020). Long-Term Follow-Up of Patients with Nodular Lymphocyte-Predominant Hodgkin Lymphoma Treated in the HD7 to HD15 Trials: A Report From the German Hodgkin Study Group. J Clin Oncol..

[14] Paschold L, Willscher E, Bein J, Vornanen M, Eichenauer DA, Simnica D (2021). Evolutionary clonal trajectories in nodular lymphocyte-predominant Hodgkin lymphoma with high risk of transformation. Haematologica.

[15] Fan Z, Natkunam Y, Bair E, Tibshirani R, Warnke RA (2003). Characterization of variant patterns of nodular lymphocyte predominant Hodgkin lymphoma with immunohistologic and clinical correlation. Am J Surg Pathol.

[16] Hartmann S, Eichenauer DA, Plütschow A, Mottok A, Bob R, Koch K (2013). The prognostic impact of variant histology in nodular lymphocyte-predominant Hodgkin lymphoma: a report from the German Hodgkin Study Group (GHSG). Blood.

[17] Hartmann S, Eichenauer DA (2020). Nodular lymphocyte predominant Hodgkin lymphoma: pathology, clinical course and relation to T-cell/histiocyte rich large B-cell lymphoma. Pathology.

[18] Xia D, Sayed S, Moloo Z, Gakinya SM, Mutuiri A, Wawire J (2022). Geographic variability of nodular lymphocyte-predominant Hodgkin Lymphoma. Am J Clin Pathol.

[19] El Weshi A, Akhtar S, Mourad WA, Ajarim D, Abdelsalm M, Khafaga Y (2007). T-cell/histiocyte-rich B-cell lymphoma: Clinical presentation, management and prognostic factors: Report on 61 patients and review of literature. Leuk Lymphoma..

[20] Bouabdallah R, Mounier N, Guettier C, Molina T, Ribrag V, Thieblemont C (2003). T-Cell/Histiocyte-Rich Large B-Cell Lymphomas and Classical Diffuse Large B-Cell Lymphomas Have Similar Outcome After Chemotherapy: A Matched-Control Analysis. J Clin Oncol..

[21] Franke S, Wlodarska I, Maes B, Vandenberghe P, Delabie J, Hagemeijer A (2001). Lymphocyte predominance Hodgkin disease is characterized by recurrent genomic imbalances. Blood.

[22] Franke S, Wlodarska I, Maes B, Vandenberghe P, Achten R, Hagemeijer A (2002). Comparative genomic hybridization pattern distinguishes T-cell/histiocyte-rich B-cell lymphoma from nodular lymphocyte predominance Hodgkin’s lymphoma. Am J Pathol.

[23] Goltsev Y, Samusik N, Kennedy-Darling J, Bhate S, Hale M, Vazquez G (2018). Deep Profiling of Mouse Splenic Architecture With CODEX Multiplexed Imaging. Cell.

[24] Schürch CM, Bhate SS, Barlow GL, Phillips DJ, Noti L, Zlobec I (2020). Coordinated Cellular Neighborhoods Orchestrate Antitumoral Immunity at the Colorectal Cancer Invasive Front. Cell.

[25] Phillips D, Matusiak M, Gutierrez BR, Bhate SS, Barlow GL, Jiang S (2021). Immune Cell Topography Predicts Response to PD-1 Blockade in Cutaneous T Cell Lymphoma. Nat Commun.

[26] Phillips D, Schürch CM, Khodadoust MS, Kim YH, Nolan GP, Jiang S (2021). Highly Multiplexed Phenotyping of Immunoregulatory Proteins in the Tumor Microenvironment by CODEX Tissue Imaging. Front Immunol.

[27] Brune V, Tiacci E, Pfeil I, Döring C, Eckerle S, van Noesel CJ (2008). Origin and pathogenesis of nodular lymphocyte-predominant Hodgkin lymphoma as revealed by global gene expression analysis. J Exp Med.

[28] Du Z, Lin JR, Rashid R, Maliga Z, Wang S, Aster JC (2019). Qualifying antibodies for image-based immune profiling and multiplexed tissue imaging. Nat Protoc.

[29] Spidlen J, Moore W, Parks D, Goldberg M, Bray C, Bierre P (2010). Data File Standard for Flow Cytometry, version FCS 3.1. Cytom A.

[30] Satija R, Farrell JA, Gennert D, Schier AF, Regev A (2015). Spatial reconstruction of single-cell gene expression data. Nat Biotechnol.

[31] Butler A, Hoffman P, Smibert P, Papalexi E, Satija R. Integrating single-cell transcriptomic data across different conditions, technologies, and species. Nature Biotechnol. 2018;36:411–20. 10.1038/nbt.4096 PMC6700744 29608179

[32] Stuart T, Butler A, Hoffman P, Hafemeister C, Papalexi E, Mauck WM (2019). Comprehensive Integration of Single-Cell Data. Cell..

[33] Hao Y, Hao S, Andersen-Nissen E, Mauck WM, Zheng S, Butler A (2021). Integrated analysis of multimodal single-cell data. Cell..

[34] Zhang X, Tsang P (2018). 196 IMP3/KOC1, a new immunohistochemical marker for differentiating classical Hodgkin Lymphoma and nodular lymphocyte predominant Hodgkin lymphoma from diffuse large B-Cell lymphoma. Am J Clin Pathol.

[35] Kosari F, Bakhshi T, Ameli F, Mokhtari M (2022). The utility of IMP3 immunohistochemical staining in differentiating nodular lymphocyte predominant Hodgkin Lymphoma from T-Cell/Histiocyte-Rich large B-Cell lymphoma. BMC Cancer.

[36] Poppe B, De Paepe P, Michaux L, Dastugue N, Bastard C, Herens C (2005). PAX5/IGH rearrangement is a recurrent finding in a subset of aggressive B-NHL with complex chromosomal rearrangements. Genes Chromosomes Cancer.

[37] Hartmann S, Döring C, Vucic E, Chan FC, Ennishi D, Tousseyn T (2015). Array comparative genomic hybridization reveals similarities between nodular lymphocyte predominant Hodgkin lymphoma and T cell/histiocyte rich large B cell lymphoma. Br J Haematol.

[38] Hartmann S, Schuhmacher B, Rausch T, Fuller L, Döring C, Weniger M (2016). Highly recurrent mutations of SGK1, DUSP2 and JUNB in nodular lymphocyte predominant Hodgkin lymphoma. Leukemia.

[39] Song JY, Egan C, Bouska AC, Zhang W, Gong Q, Venkataraman G (2020). Genomic characterization of diffuse large B-cell lymphoma transformation of nodular lymphocyte-predominant Hodgkin lymphoma. Leukemia.

[40] Schuhmacher B, Bein J, Rausch T, Benes V, Tousseyn T, Vornanen M (2019). JUNB, DUSP2, SGK1, SOCS1 and CREBBP are frequently mutated in T-cell/histiocyte-rich large B-cell lymphoma. Haematologica.

[41] Bein J, Thurner L, Hansmann ML, Hartmann S (2020). Lymphocyte predominant cells of nodular lymphocyte predominant Hodgkin lymphoma interact with rosetting T cells in an immunological synapse. Am J Hematol.

[42] Veldman J, Alsada ZND, van den Berg A, Plattel WJ, Diepstra A, Visser L (2021). Soluble PD-L1 is a promising disease biomarker but does not reflect tissue expression in classic Hodgkin lymphoma. Br J Haematol.

[43] Nam-Cha SH, Roncador G, Sanchez-Verde L, Montes-Moreno S, Acevedo A, Domínguez-Franjo P (2008). PD-1, a follicular T-cell marker useful for recognizing nodular lymphocyte-predominant Hodgkin lymphoma. Am J Surg Pathol.

[44] Churchill HR, Roncador G, Warnke RA, Natkunam Y (2010). Programmed death 1 expression in variant immunoarchitectural patterns of nodular lymphocyte predominant Hodgkin lymphoma: comparison with CD57 and lymphomas in the differential diagnosis. Hum Pathol.

[45] Visser L, Rutgers B, Diepstra A, van den Berg A, Sattarzadeh A (2016). Characterization of the microenvironment of nodular lymphocyte predominant Hodgkin lymphoma. Int J Mol Sci.

[46] Mita Y, Kimura MY, Hayashizaki K, Koyama-Nasu R, Ito T, Motohashi S (2018). Crucial role of CD69 in anti-tumor immunity through regulating the exhaustion of tumor-infiltrating T cells. Int Immunol.

[47] Younes S, Rojansky RB, Menke JR, Gratzinger D, Natkunam Y (2021). Pitfalls in the Diagnosis of Nodular Lymphocyte Predominant Hodgkin Lymphoma: Variant Patterns, Borderlines and Mimics. Cancers.

[48] Eladl AE, Satou A, Elsayed AA, Suzuki Y, Shimizu-Kohno K, Kato S (2015). Nodular lymphocyte predominant Hodgkin lymphoma: Clincopathological study of 25 cases from Japan with a reappraisal of tissue associated macrophages. Pathol Int.

[49] Autio M, Leivonen SK, Brück O, Karjalainen-Lindsberg ML, Pellinen T, Leppä S (2022). Clinical Impact of Immune Cells and Their Spatial Interactions in Diffuse Large B-Cell Lymphoma Microenvironment. Clin Cancer Res.

[50] Chemnitz JM, Eggle D, Driesen J, Classen S, Riley JL, Debey-Pascher S (2007). RNA fingerprints provide direct evidence for the inhibitory role of TGF and PD-1 on CD4 T cells in Hodgkin lymphoma. Blood.

[51] Vermare A, Guérin MV, Peranzoni E, Bercovici N (2022). Dynamic CD8+ T Cell Cooperation with Macrophages and Monocytes for Successful Cancer Immunotherapy. Cancers.

[52] Rahemtullah A, Harris NL, Dorn ME, Preffer FI, Hasserjian RP (2008). Beyond the lymphocyte predominant cell: CD4+CD8+ T-cells in nodular lymphocyte predominant Hodgkin lymphoma. Leuk Lymphoma.

[53] Hartmann S, Soltani AS, Bankov K, Bein J, Hansmann ML, Rosenwald A (2022). Tumour cell characteristics and microenvironment composition correspond to clinical presentation in newly diagnosed nodular lymphocyte-predominant Hodgkin lymphoma. Br J Hematol.

[54] Sadeghi Shoreh Deli A, Scharf S, Steiner Y, Bein J, Hansmann ML, Hartmann S (2022). 3D analyses reveal T cells with activated nuclear features in T-cell/histiocyte-rich large B-cell lymphoma. Mod Pathol.

