# Supplementary material for: Spatial phenotyping of nodular lymphocyte predominant Hodgkin lymphoma and T-cell/histiocyte-rich large B-cell lymphoma
Source: Blood Cancer J. 2024 May 31;14(1):92. doi: 10.1038/s41408-024-01073-z (PMC11143196; doi:10.1038/s41408-024-01073-z)
Supplement: Supplementary file 1 — Supplemental figures [file 41408_2024_1073_MOESM1_ESM.docx]

**Younes et al., Supplementary figures**

**Spatial Phenotyping of Nodular Lymphocyte Predominant Hodgkin Lymphoma and**

**T-cell/Histiocyte-Rich Large B-cell Lymphoma Reveals Distinct Cellular Interactions**

**Contents:**

**Supplementary Figure 1: Staining profiles of individual markers in CODEX panel**

**Supplementary Figure 2: Segmentation overlay using Mav software**

**Supplementary Figure 3: Regions of interest in NLPHL and THRLBCL**

**Supplementary figure 4: Cell type bar chart by case**

**Supplementary Figure 5: Corr plots for spatial interactions between various cellular populations across the studied groups**

**Supplementary Figure 6: ROIs, population frequencies and spatial interactions for representative cases across studied groups**

**Supplementary Figure 7: Qupath measurements data**

**Supplementary Figure 1: Staining profiles of individual markers in CODEX panel**

**
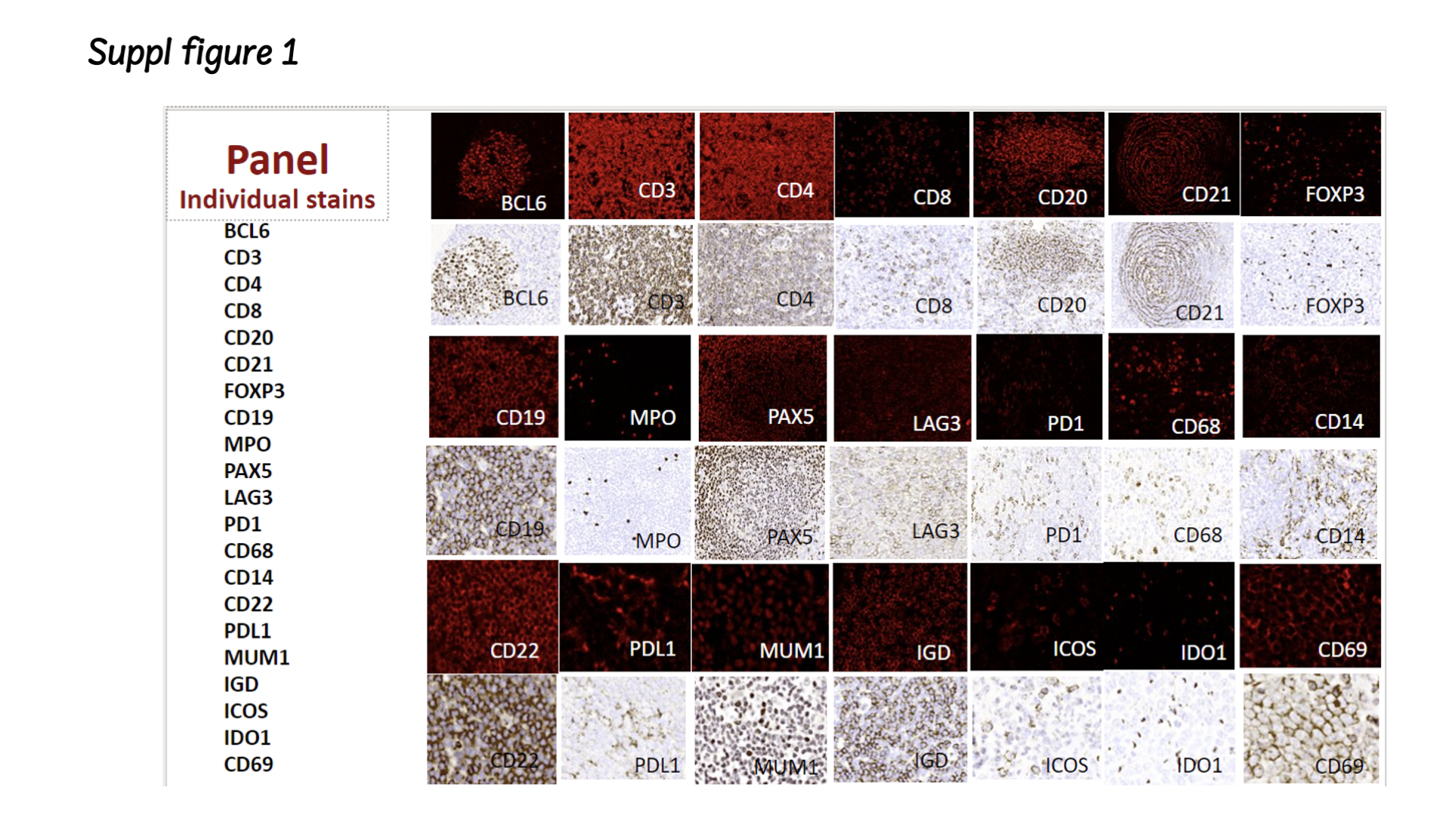
**

**Supplementary Figure 2: Segmentation overlay using Mav software**


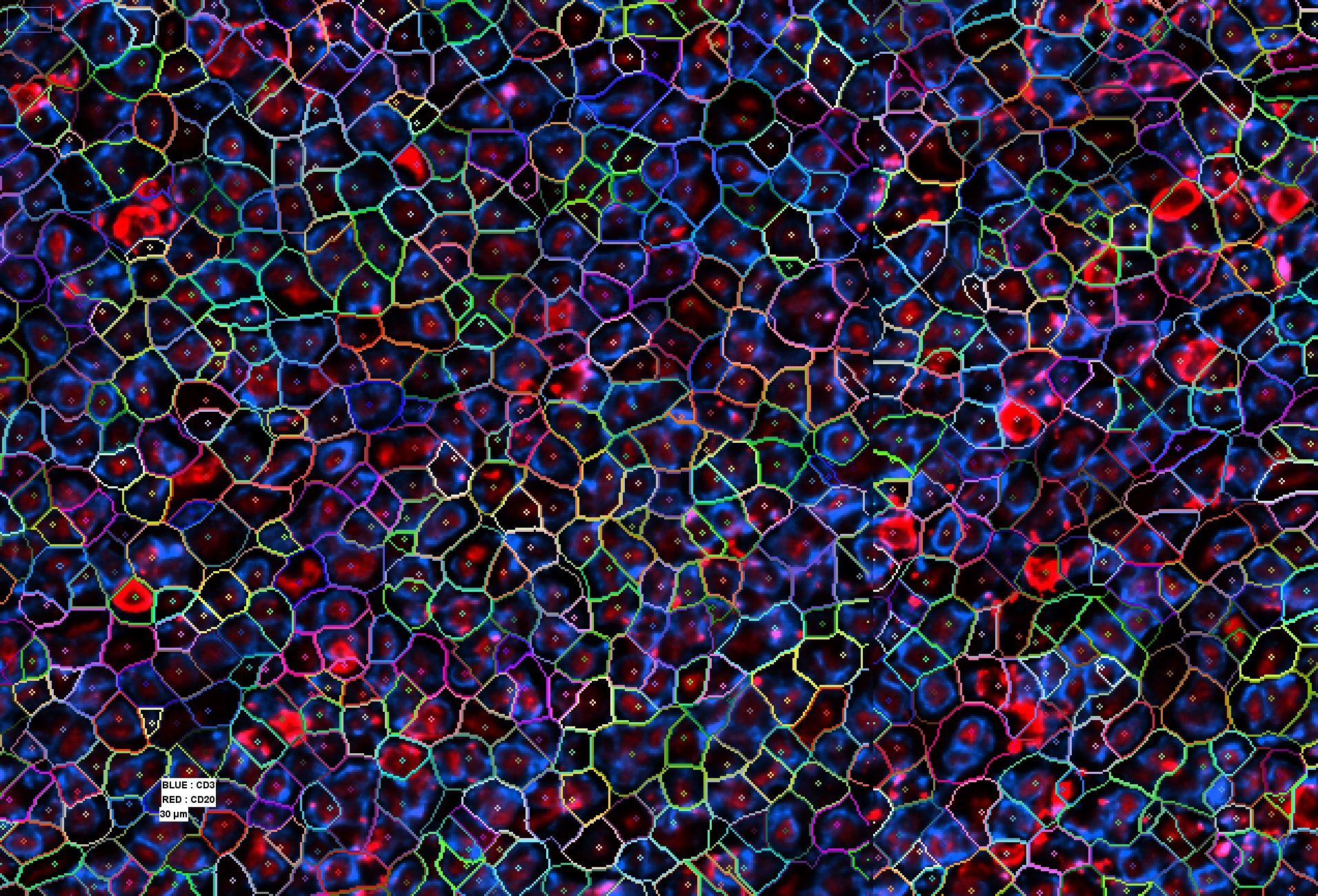


**Supplementary Figure 3: Regions of interest in NLPHL and THRLBCL**


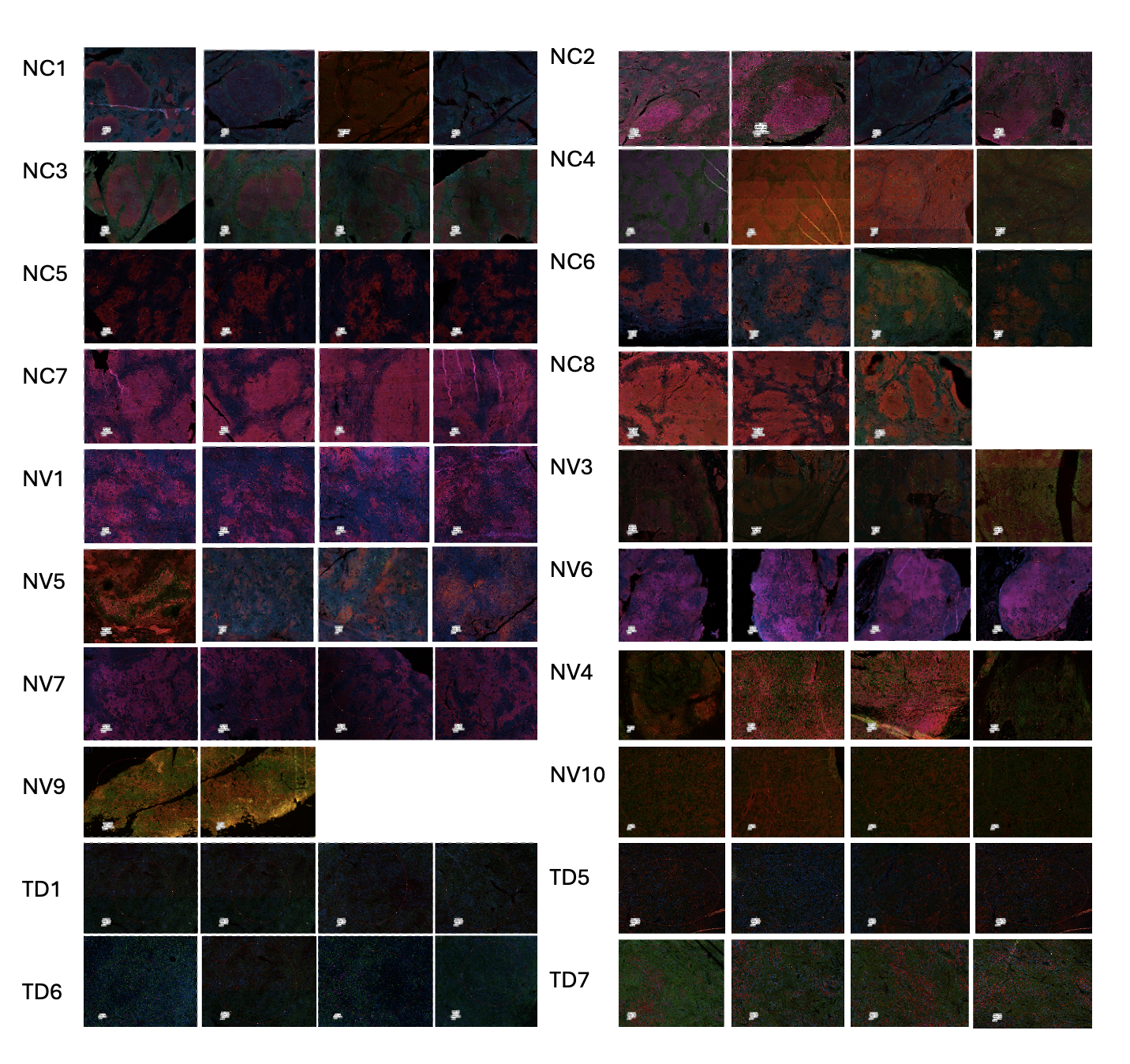


**Supplementary figure 4: Cell type bar chart by case**


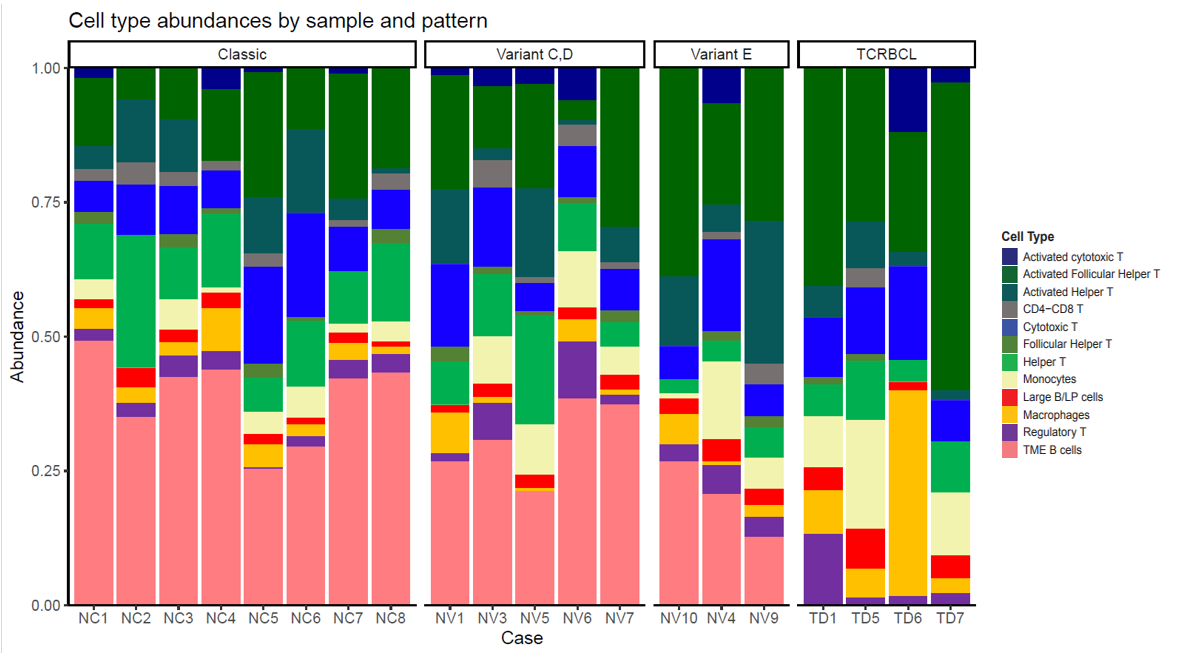


**Supplementary Figure 5: Corr plots for spatial interactions between various cellular populations across the studied groups**

**Supplementary Figure 6: ROIs, population frequencies and spatial interactions for representative cases across studied groups**

**
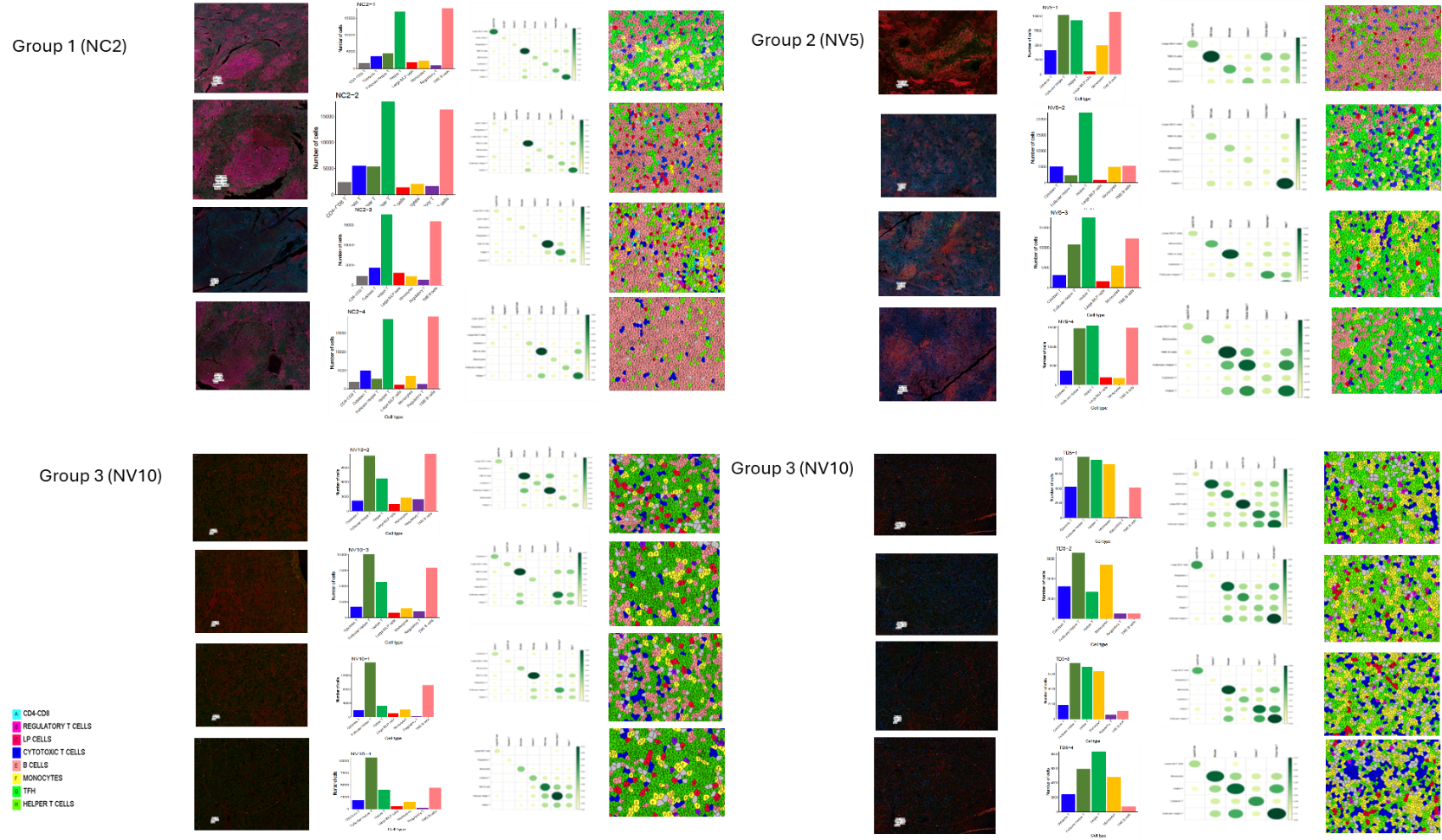
**

**Supplementary Figure 7: Qupath measurements data**
