# Supplementary material for: Spatial phenotyping of nodular lymphocyte predominant Hodgkin lymphoma and T-cell/histiocyte-rich large B-cell lymphoma
Source: Blood Cancer J. 2024 May 31;14(1):92. doi: 10.1038/s41408-024-01073-z (PMC11143196; doi:10.1038/s41408-024-01073-z)
Supplement: Supplementary file 2 — Supplemental Tables [file 41408_2024_1073_MOESM2_ESM.docx]

**Younes et al., Supplemental Tables**

**Spatial Phenotyping of Nodular Lymphocyte Predominant Hodgkin Lymphoma and**

**T-cell/Histiocyte-Rich Large B-cell Lymphoma Reveals Distinct Cellular Interactions**

**Contents:**

**Supplemental Table 1: Antibody information for CODEX and immunohistochemistry**

**Supplemental Table 2: Cell cycle data**

**Supplemental Table 3: Z-scores by case**

**Supplemental Table 4: Macrophage/monocyte markers expression by immunohistochemistry**

**Supplemental Table 5: Interaction counts among cell types in group 1 NLPHL cases**

**Supplemental Table 6: Interaction counts among cell types in group 2 NLPHL cases**

**Supplemental Table 7: Interaction counts among cell types in group 3 NLPHL cases**

**Supplemental Table 8: Interaction counts among cell types in THRLBCL cases**

**Supplemental Table 9: Summary data of population frequencies and unique spatial interactions**

**Supplemental Table 10: Nuclear area and perimeter measurements in NLPHL and THRLBCL**

**Supplemental Table 1: Antibody information for CODEX and immunohistochemistry**

| **Antibody** | **Barcode** | **Clones** | **Vendor** |
| --- | --- | --- | --- |
| **CODEX** | | | |
| CD69 | BX017 | EPR21814 | Abcam, Cambridge, UK |
| IgD | BX046 | EPR6146 | Abcam, Cambridge, UK |
| PAX5 | BX021 | D7H5X | Cell Signaling Technology, Danvers, MA |
| MUM1 | BX024 | EP5699 | Abcam, Cambridge, UK |
| CD19 | BX043 | LE-CD19 | Bio-Rad Labrotories, Hercules, CA |
| CD22 | BX034 | EPR20061 | Abcam, Cambridge, UK |
| MPO | BX006 | EPR20257 | Abcam, Cambridge, UK |
| CD14 | BX030 | EPR3653 | Abcam, Cambridge, UK |
| BCL6 | BX023 | IG191E/A8 | BioLegend, San Diego, CA |
| CD223/LAG3 | BX027 | EPR4392 (2) | Abcam, Cambridge, UK |
| CD274/PDL1 | BX041 | CAL10 | Abcam, Cambridge, UK |
| PD1 | BX014 | D4W2J | Cell Signaling Technology, Danvers, MA |
| FOXP3 | BX020 | 236A/E7 | Invitrogen, Carlsbad, CA |
| ICOS | BX042 | SP98 | Abcam, Cambridge, UK |
| IDO1 | BX025 | D5J4E | Cell Signaling Technology, Danvers, MA |
| CD4 | Bx003 | EPR6855 | Akoya Biosciences, Menlo Park, CA |
| CD8 | Bx026 | C8/144B | Akoya Biosciences, Menlo Park, CA |
| CD20 | BX007 | SP32 | Akoya Biosciences, Menlo Park, CA |
| CD21 | Bx032 | EP3093 | Akoya Biosciences, Menlo Park, CA |
| CD68 | BX015 | KP1 | Akoya Biosciences, Menlo Park, CA |
| CD3e | BX045 | EP449E | Akoya Biosciences, Menlo Park, CA |
| **IMMUNOHISTOCHEMISTRY** | | | |
| CD20 | - | L26 | Benchmark Ultra, Roche Tissue Diagnostics, Tucson, AZ |
| MEF2B | - | Polyclonal | Leica-Bond III, Leica Biosystems, Buffalo Grove, IL |
| PU.1 | - | G148-74 | Leica-Bond III, Leica Biosystems, Buffalo Grove, IL |
| CD163 | - | MRQ-26 | Benchmark Ultra, Roche Tissue Diagnostics, Tucson, AZ |
| CD14 | - | EPR3653 | Leica-Bond III, Leica Biosystems, Buffalo Grove, IL |

**Supplemental Table 2: Cell cycle data**

| **Channel 1** | **Exposure Time** | **Alexa 488** | **Oligo Barcode** | **Clone** | **Antibody Dilution** | **Exposure Time** | **Atto 550** | **Oligo Barcode** | **Clone** | **Antibody Dilution** | **Exposure Time** | **Alexa 647** | **Oligo Barcode** | **Clone** | **Antibody Dilution** | **Exposure Time** |
| --- | --- | --- | --- | --- | --- | --- | --- | --- | --- | --- | --- | --- | --- | --- | --- | --- |
| Hoechst | 20 | Blank |  |  |  | 400 | Blank |  |  |  | 400 | Blank |  |  |  | 500 |
| Hoechst | 20 | IgD | BX046 | EPR6146 | 1:25 | 400 | Bcl6 | BX023 | IG191E/A8 | 1:50 | 400 | CD3 | BX045 | EP449E | 1:50 | 500 |
| Hoechst | 20 | CD19 | BX043 | LE-CD19 | 1:50 | 400 | CD20 | BX035 | SP32 | 1:50 | 400 | CD4 | BX003 | EPR6855 | 1:50 | 500 |
| Hoechst | 20 | CD22 | BX034 | EPR20061 | 0.1111111 | 400 | CD21 | BX032 | EP3093 | 0.1805556 | 400 | CD14 | BX030 | EPR3653 | 1:50 | 500 |
| Hoechst | 20 | IDO1 | BX025 | D5J4E | 1:50 | 400 | CD69 | BX017 | EPR21814 | 1:50 | 400 | CD68 | BX015 | KP1 | 1:50 | 500 |
| Hoechst | 20 | Empty |  | - |  | 400 | Foxp3 | BX020 | 236A/E7 | 1:50 | 400 | ICOS | BX042 | SP98 | 1:50 | 500 |
| Hoechst | 20 | Empty |  | - |  | 400 | PD1 | BX014 | D4W2J | 1:50 | 400 | LAG3 | BX027 | EPR4392 (2) | 1:50 | 500 |
| Hoechst | 20 | Empty |  | - |  | 400 | PDL1 | BX041 | CAL10 | 1:25 | 400 | MPO | BX006 | EPR20257 | 0.11111 | 500 |
| Hoechst | 20 | Empty |  | - |  | 400 | CD8 | BX026 | C8/144B | 1:50 | 400 | MUM1 | BX024 | EP5699 | 1:50 | 500 |
| Hoechst | 20 | Empty |  | - |  | 400 | Empty | - |  |  | 400 | PAX5 | BX021 | D7H5X | 1:25 | 500 |
| Hoechst | 20 | Blank |  |  |  | 400 | Blank |  |  |  | 400 | Blank |  |  |  | 500 |

**Supplemental Table 3: Z-scores by case**

| **Z SCORES** | **NC1** | **NC2** | **NC3** | **NC4** | **NC5** | **NC6** | **NC7** | **NV1** | **NV10** | **NC8** | **NV3** | **NV4** | **NV5** | **NV6** | **NV7** | **NV9** | **TD1** | **TD5** | **TD6** | **TD7** |
| --- | --- | --- | --- | --- | --- | --- | --- | --- | --- | --- | --- | --- | --- | --- | --- | --- | --- | --- | --- | --- |
| Large B/LP cells (CD20/BCL6) | -0.67 | 0.60 | -0.12 | 0.13 | -0.56 | -1.03 | -0.41 | -0.82 | 0.21 | -1.36 | -0.19 | 0.72 | -0.19 | -0.57 | -0.02 | 0.28 | 0.79 | 3.16 | -1.08 | 1.12 |
| TME B cells (CD20/IgD) | 1.81 | 0.23 | 1.15 | 1.21 | -0.72 | -0.26 | 1.08 | -0.37 | -0.47 | 0.33 | -0.22 | -1.29 | -1.18 | 0.17 | 0.39 | -1.84 | NA | NA | NA | NA |
| Helper T (CD3/CD4) | 0.19 | 2.75 | 0.08 | 0.82 | -0.62 | 0.50 | 0.11 | -0.18 | -1.28 | 0.44 | 0.34 | -1.14 | 1.77 | -0.33 | -0.94 | -0.68 | -0.77 | 0.19 | -1.24 | -0.01 |
| Activated Helper T (CD3/CD4/CD69/LAG3) | -0.57 | 0.46 | 0.26 | NA | 0.26 | 1.04 | -0.60 | 0.93 | 0.69 | -1.11 | -0.91 | -0.54 | 1.03 | -1.11 | -0.29 | 2.80 | -0.45 | -0.01 | -0.95 | -0.95 |
| Follicular Helper T (CD3/CD4/PD1/BCL6) | 0.95 | NA | 1.19 | -1.02 | 1.23 | -1.33 | NA | 1.55 | NA | 0.68 | -0.60 | -0.12 | -1.21 | -1.01 | 0.55 | 0.55 | -0.62 | -0.80 | NA | NA |
| Activated Follicular Helper T (CD3/CD4/PD1/BCL6/CD69/LAG3) | -0.63 | -1.21 | -0.88 | -0.57 | 0.12 | -0.76 | 0.21 | 0.10 | 1.43 | -0.46 | -0.78 | -0.31 | -0.22 | -1.39 | 0.58 | 0.65 | 1.24 | 0.47 | -0.38 | 2.79 |
| Cytotoxic T (CD3/CD8) | -0.99 | -0.24 | -0.23 | -0.69 | 1.69 | 2.09 | -0.42 | 1.40 | -0.93 | -0.96 | 0.93 | 1.27 | -1.23 | -0.41 | -0.62 | -0.95 | -0.02 | 0.38 | 0.56 | -0.64 |
| Activated cytotoxic T (CD3/CD8/CD69/LAG3) | -0.65 | NA | NA | 0.21 | -1.13 | NA | -0.98 | -0.85 | NA | NA | -0.04 | 1.05 | -0.25 | 0.75 | NA | NA | NA | NA | 2.18 | -0.30 |
| Regulatory T (CD3/CD4/FOXP3) | -0.49 | -0.37 | 0.13 | -0.02 | -1.16 | -0.57 | -0.04 | -0.70 | -0.17 | -0.32 | 1.01 | 0.43 | NA | 1.93 | -0.67 | 0.06 | 2.92 | -0.75 | -0.81 | -0.43 |
| CD4-CD8 T (CD3/CD4/CD8) | -0.29 | 1.24 | 0.11 | -0.62 | -0.13 | NA | -1.10 | NA | NA | -0.08 | 1.91 | -1.06 | -1.24 | 0.76 | -1.16 | 1.09 | NA | 0.58 | NA | NA |
| Macrophage (CD68) | -0.13 | -0.31 | -0.36 | 0.57 | -0.10 | -0.42 | -0.25 | 0.55 | 0.17 | -0.58 | -0.62 | -0.69 | -0.72 | -0.18 | -0.62 | -0.40 | 0.46 | 0.07 | 3.90 | -0.34 |
| Monocyte (CD68/MPO/CD14) | -0.62 | NA | -0.21 | -1.22 | -0.59 | -0.22 | -1.10 | NA | -1.22 | -0.79 | 0.33 | 1.34 | 0.40 | 0.51 | -0.40 | -0.17 | 0.38 | 2.59 | NA | 0.99 |

**Supplemental Table 4: Macrophage/monocyte marker expression by immunohistochemistry**

| **Marker** | **Case Type** | **Mean ± SD** | **Median** | **Mann-Whitney U (P)** |
| --- | --- | --- | --- | --- |
| PU.1 | THRLBCL | 52.3 ± 28.9 | 55.3 | 70.5 (0.009) |
|  | NLPHL, Pattern E | 27.26 ± 17.2 | 21.9 |  |
| CD163 | THRLBCL | 55.8 ± 34.9 | 73.22 | 42.00 (<0.001) |
|  | NLPHL, Pattern E | 13.0 ± 17.2 | 3.5 |  |
| CD14 | THRLBCL | 45.6 ± 27.0 | 61.2 | 67.00 (0.007) |
|  | NLPHL, Pattern E | 19.9 ± 16.1 | 13.1 |  |

**Supplemental Table 5: Interaction counts among cell types in group 1 NLPHL cases**

| **GROUP 1 INTERACTIONS** | **TFH CELLS** | **REGULATORY T-CELLS** | **LP CELLS** | **B-CELLS** | **MACRO**  **PHAGES** | **CD4/CD8 T-CELLS** | **CYTOTOXIC T-CELLS** | **HELPER T-CELLS** |
| --- | --- | --- | --- | --- | --- | --- | --- | --- |
| TFH CELLS | 0.485 | 0.038 | 0.094 | 0.264 | 0.091 | 0.045 | 0.136 | 0.158 |
| REGULATORY T-CELLS | 0.038 | 0.216 | 0.030 | 0.039 | 0.069 | 0.081 | 0.082 | 0.099 |
| LP CELLS | 0.094 | 0.030 | 0.260 | 0.065 | 0.040 | 0.028 | 0.047 | 0.056 |
| B CELLS | 0.264 | 0.039 | 0.065 | 1.029 | 0.100 | 0.043 | 0.161 | 0.294 |
| MACROPHAGES | 0.091 | 0.069 | 0.040 | 0.100 | 0.355 | 0.072 | 0.132 | 0.151 |
| CD4/CD8 T-CELLS | 0.045 | 0.081 | 0.028 | 0.043 | 0.072 | 0.210 | 0.147 | 0.141 |
| CYTOTOXIC T-CELLS | 0.136 | 0.082 | 0.047 | 0.161 | 0.132 | 0.147 | 0.399 | 0.214 |
| HELPER T-CELLS | 0.158 | 0.099 | 0.056 | 0.294 | 0.151 | 0.141 | 0.214 | 0.580 |

**Supplemental Table 6: Interaction counts among cell types in group 2 NLPHL cases**

| **GROUP 2 INTERACTIONS** | **REGULATORY T-CELLS** | **LP CELLS** | **TFH CELLS** | **CYTOTOXIC T-CELLS** | **MACRO**  **PHAGES** | **B-CELLS** | **HELPER T-CELLS** | **CD4/CD8 T-CELLS** |
| --- | --- | --- | --- | --- | --- | --- | --- | --- |
| REGULATORY T-CELLS | 0.233 | 0.030 | 0.067 | 0.107 | 0.063 | 0.091 | 0.063 | 0.109 |
| LP CELLS | 0.030 | 0.241 | 0.058 | 0.035 | 0.023 | 0.062 | 0.045 | 0.034 |
| TFH CELLS | 0.067 | 0.058 | 0.575 | 0.177 | 0.112 | 0.294 | 0.258 | 0.051 |
| CYTOTOXIC T-CELLS | 0.107 | 0.035 | 0.177 | 0.355 | 0.111 | 0.189 | 0.193 | 0.126 |
| MACROPHAGES | 0.063 | 0.023 | 0.112 | 0.111 | 0.345 | 0.086 | 0.120 | 0.086 |
| B-CELLS | 0.091 | 0.062 | 0.294 | 0.189 | 0.086 | 0.807 | 0.210 | 0.066 |
| HELPER T-CELLS | 0.063 | 0.045 | 0.258 | 0.193 | 0.120 | 0.210 | 0.515 | 0.055 |
| CD4/CD8 T-CELLS | 0.109 | 0.034 | 0.051 | 0.126 | 0.086 | 0.066 | 0.055 | 0.258 |

**Supplemental Table 7: Interaction counts among cell types in group 3 NLPHL cases**

| **GROUP 3 INTERACTIONS** | **LP CELLS** | **REGULATORY T-CELLS** | **B-CELLS** | **CYTOTOXIC T-CELLS** | **TFH CELLS** | **MACRO PHAGES** | **HELPER**  **T-CELLS** | **CD4/CD8**  **T-CELLS** |
| --- | --- | --- | --- | --- | --- | --- | --- | --- |
| LPCELLS | 0.292 | 0.029 | 0.048 | 0.044 | 0.079 | 0.040 | 0.047 | 0.034 |
| REGULATORY T-CELLS | 0.029 | 0.186 | 0.094 | 0.073 | 0.076 | 0.043 | 0.093 | 0.050 |
| B-CELLS | 0.048 | 0.094 | 0.657 | 0.175 | 0.262 | 0.070 | 0.223 | 0.042 |
| CYTOTOXIC T-CELLS | 0.044 | 0.073 | 0.175 | 0.414 | 0.161 | 0.131 | 0.131 | 0.086 |
| TFH CELLS | 0.079 | 0.076 | 0.262 | 0.161 | 0.583 | 0.116 | 0.253 | 0.064 |
| MACROPHAGES | 0.040 | 0.043 | 0.070 | 0.131 | 0.116 | 0.361 | 0.082 | 0.037 |
| HELPER T-CELLS | 0.047 | 0.093 | 0.223 | 0.131 | 0.253 | 0.082 | 0.367 | 0.080 |
| CD4/CD8 T-CELLS | 0.034 | 0.050 | 0.042 | 0.086 | 0.064 | 0.037 | 0.080 | 0.182 |

**Supplemental Table 8: Interaction count between cell types in THRLBCL cases**

| **GGROUP 4**  **INTERACTIONS** | **LP CELLS** | **MACROPHAGES** | **TFH CELLS** | **HELPER T-CELLS** | **CYTOTOXIC**  **T-CELLS** | **REGULATORY**  **T-CELLS** |
| --- | --- | --- | --- | --- | --- | --- |
| LP CELLS | 0.509 | 0.046 | 0.075 | 0.095 | 0.043 | 0.027 |
| MACROPHAGES | 0.046 | 0.423 | 0.224 | 0.182 | 0.165 | 0.048 |
| TFH CELLS | 0.075 | 0.224 | 0.669 | 0.259 | 0.175 | 0.122 |
| HELPER T-CELLS | 0.095 | 0.182 | 0.259 | 0.420 | 0.145 | 0.070 |
| CYTOTOXIC T-CELLS | 0.043 | 0.165 | 0.175 | 0.145 | 0.370 | 0.051 |
| REGULATORY T-CELLS | 0.027 | 0.048 | 0.122 | 0.070 | 0.051 | 0.247 |

**Supplemental Table 9: Summary data of population frequencies and unique spatial interactions**

| **Population** | **Group 1** | | **Group 2** | | **Group 3** | | **THRLBCL** | |
| --- | --- | --- | --- | --- | --- | --- | --- | --- |
|  | **Frequency** | **Unique spatial interactions** | **Frequency** | **Unique spatial interactions** | **Frequency** | **Unique spatial interactions** | **Frequency** | **Unique spatial interactions** |
| LP/tumor cells ​ | low | TFH, TME B-cells | low | TME B-cells, TFH | high | TFH, TME B-cells | The highest | TFH, helper T-cells |
| TME B-cells*​ | The highest number | **LP cells, Helper T-cells and T-reg** | More & close to LP​ | TFH, LP cells | lower | LP cells | Not detected |  |
| Helper T-cells*​ | The highest number | **TME B-cells**, **cytotoxic T-cells** and **monocytes** | High​ | **Cytotoxic T-cells** | Lower |  | Lower | **Monocytes**, tumor cells |
| Activated Helper T-cells*​ | High​ |  | High​ |  | High​ |  | Low |  |
| TFH​ cells | High  ​ | LP cells | High | LP cells, TME B-cells, and **cytotoxic T-cells, T-reg** | High | **Cytotoxic, T-reg, LP cells** | low | **monocytes,** tumor cells, T-reg, Cytotoxic |
| Activated TFH*​ cells | The lowest number |  | low |  | high​ |  | High​ |  |
| Treg*​ cells | Low | **TME B-cells**, **cytotoxic,** **monocytes, CD4-CD8 positive T-cells** | High | **cytotoxic T-cells, TFH** | High | **TFH** | High​ | TFH |
| CD4-CD8 positive T-cells | Detected | **Monocytes, T-reg, and cytotoxic T-cells** | Detected |  | Detected |  |  |  |
| Cytotoxic T-cells | Low | **Helper T-cells** and **CD4-CD8 positive T-cells, T-reg** | low | **Helper T-cells,** **TFH**, and **T-reg cels** | low | TFH | High | **Monocytes, TFH** |
| Activated cytotoxic T-cells | Low |  | low |  | Low |  | High |  |
| Macrophages | Low | **Helper T-cells** and **CD4-CD8 positive T-cells, T-reg** | low |  | High |  | The highest | **Helper T-cells**, **cytotoxic T-cells**, **TFH** |
| Monocytes | Low |  | low |  | High |  | The highest |  |

**Supplemental Table 10: Nuclear area and perimeter measurements in NLPHL and THRLBCL**

| **Property** | **Group and Feature** | **Min.** | **1st Qu.** | **Median** | **Mean** | **3rd Qu.** | **Max.** | **SD** | **Cell Count** |
| --- | --- | --- | --- | --- | --- | --- | --- | --- | --- |
| Nucleus: perimeter | Group 1 NLPHL | 10.8098 | 14.60155 | 17.5859 | 19.33311 | 22.13053 | 94.9054 | 6.926257384 | 7910 |
|  | Group 2 NLPHL | 10.7871 | 15.0045 | 18.2311 | 19.56098 | 22.412 | 82.4607 | 6.37146433 | 8045 |
|  | Group 3 NLPHL | 10.9251 | 16.14423 | 19.91675 | 21.39549 | 25.14718 | 72.0826 | 7.014452414 | 6492 |
|  | THRLBCL | 10.8106 | 16.2968 | 19.6384 | 20.70375 | 24.10655 | 59.6871 | 6.026797576 | 4723 |
| Nucleus: Area | Group 1 NLPHL | 8 | 13.75 | 18.75 | 23.13306 | 26.75 | 356.75 | 16.14327468 | 7910 |
|  | Group 2 NLPHL | 7.75 | 15 | 20.5 | 23.97216 | 28.5 | 289.75 | 14.67151679 | 8045 |
|  | Group 3 NLPHL | 8.25 | 16.75 | 24 | 28.13355 | 34.5 | 246.25 | 16.97008087 | 6492 |
|  | THRLBCL | 7.75 | 17.5 | 24.25 | 26.87582 | 33.5 | 109 | 12.80267018 | 4723 |
